# Who Tells the Story of the Treatment of Androgenetic Alopecia?—A Bibliometric Analysis From 2003 to 2023

**DOI:** 10.1111/jocd.70370

**Published:** 2025-08-31

**Authors:** Yulin Sun, Mingyang Lu, Xuejun Gao, Yutong Liu, Jizhen Ren, Yanjin Wang, Xia Cai

**Affiliations:** ^1^ Department of Plastic Surgery Affiliated Hospital of Qingdao University Qingdao China; ^2^ Department of Plastic Surgery Shanghai East Hospital, Tongji University School of Medicine Shanghai China

**Keywords:** androgenetic alopecia, bibliometric analysis, global research collaboration, novel treatments, research trends

## Abstract

**Background:**

Androgenetic alopecia (AGA) is a common form of nonscarring hair loss that affects both men and women, significantly influencing psychological well‐being and quality of life. Although AGA accounts for over 90% of male hair loss, there has been limited bibliometric analysis to guide the direction of research and the development of treatments.

**Methods:**

A thorough literature search was performed using the Web of Science database, resulting in the identification of 2203 articles on AGA treatment published between 2003 and 2023. The search was restricted to English‐language peer‐reviewed publications. Bibliometric analysis was conducted to assess research trends, publication growth, and key contributors in AGA treatment research.

**Results:**

The United States and China emerged as the leading contributors, with major institutions such as the Mayo Clinic and Harvard Medical School making significant contributions. The research focus has shifted from traditional drug therapies to innovative treatments, such as platelet‐rich plasma (PRP), and anti‐androgen therapy. Key research areas included anti‐androgen treatments, PRP, and microneedling, whereas emerging trends pointed to stem cell therapy and gene therapy as future directions.

**Conclusion:**

The bibliometric analysis of AGA treatment research demonstrates an increasing interest in novel therapies and a transition towards more personalized and effective treatment approaches. These findings provide valuable insights into the development of AGA research and underscore areas in need of further exploration, offering a strategic guide for future research and clinical practice enhancements.

## 1 Introduction

Androgenetic alopecia (AGA) is a common non‐cicatricial, progressive condition marked by the miniaturization of hair follicles, resulting in hair loss. This condition has a genetic predisposition and is androgen‐dependent [1]. AGA can manifest at any age, affecting approximately 80% of males and 50% of females by their 70s [2]. In men, hair loss typically occurs in the temporal, frontal, and crown areas of the scalp, whereas women generally experience diffuse thinning on the top of the head [3]. Beyond its physical impact and effect on external appearance, AGA significantly affects patients' psychological well‐being, often leading to reduced self‐esteem, depression, and a diminished quality of life. Given its high prevalence and profound personal impact, there is an urgent need for treatments that minimize side effects while providing meaningful and lasting therapeutic benefits. Currently, several treatments for AGA are available, including Minoxidil, anti‐androgen therapy, platelet‐rich plasma (PRP) [4], botulinum toxin [5], and nanosystem‐based treatments [6].

However, there is still no safe, effective, and permanent cure for AGA. As research on AGA continues to advance, a growing number of novel therapeutic strategies are being introduced. Bibliometric analysis, a widely used method for analyzing large volumes of academic literature and assessing their influence in a specific field [7], can offer valuable insights into the current state of AGA research and treatment strategies, thereby enhancing clinical practice.

## 2 Methods

**FIGURE 1:**
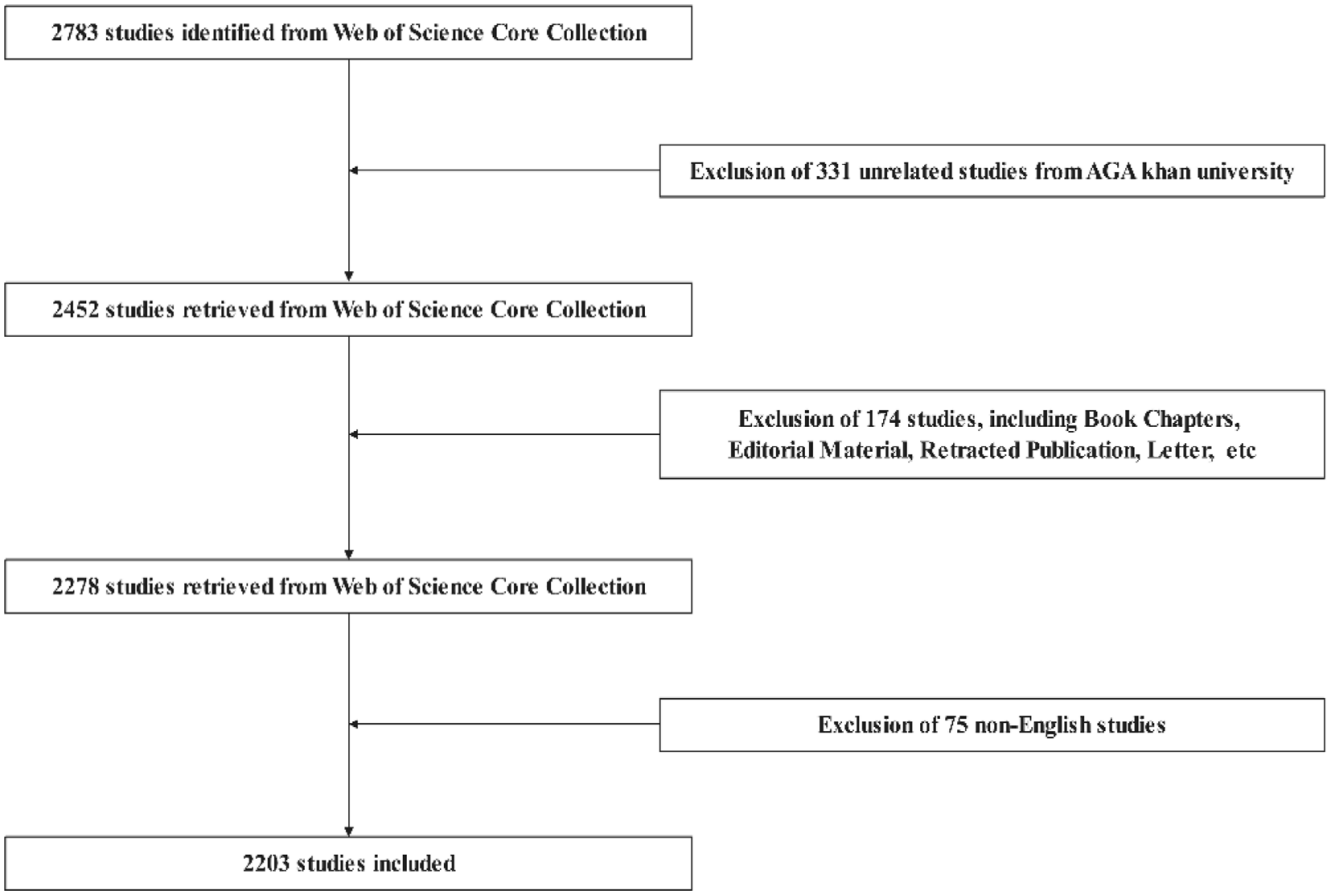
A flowchart illustrating the process of literature screening for current research on treatment methods for androgenetic alopecia (AGA).

We conducted a literature search using the Web of Science (WOS) database on May 10, 2024. The search formula was as follows: (((TS = (Therap*)) OR TS = (Therapeutic)) OR TS = (Treatment*)) AND ((((((TS = (Androgen alopecia)) OR TS = (Androgenic Alopecia)) OR TS = (Pattern Alopecia)) OR TS = (Pattern Baldness)) OR TS = (AGA))). The publication period was limited to 2003/01/01–2023/12/31, resulting in a total of 2783 articles (as shown in Figure 1). Excluded from this analysis were conference abstracts, preprints, book chapters, corrections, editorial materials, conference proceedings, withdrawn publications, data files, letters, and retractions. The language of the included research was restricted to English.

## 3 Results

### 3.1 Publication Analysis

**FIGURE 2:**
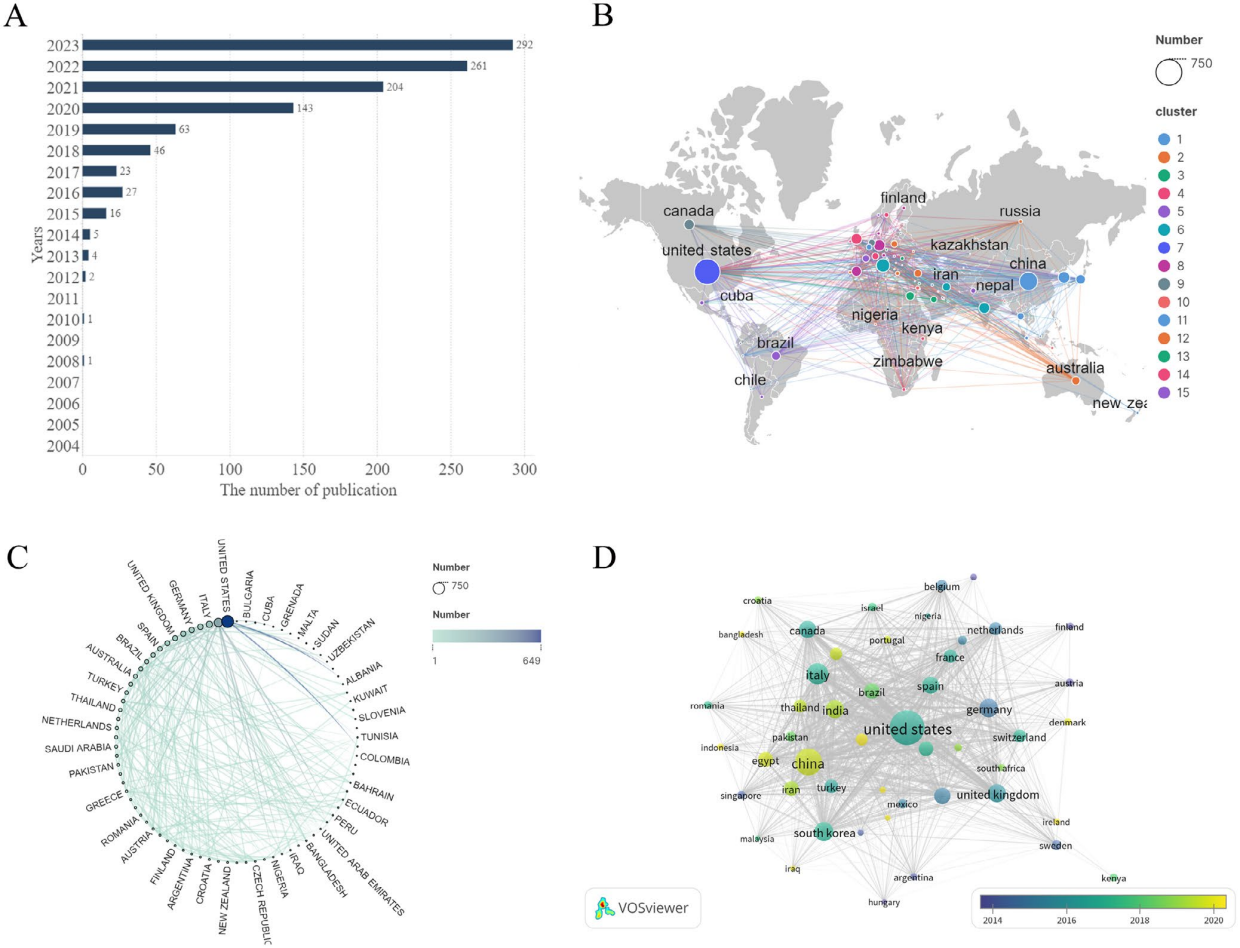
Refined global trends in research publications focused on AGA treatment. (A) A decade‐long analysis of global publication trends in AGA treatment from 2003 to 2023. (B) A geographical overview of AGA therapeutic research, highlighting international collaborations among countries based on co‐authorship analysis. (C) A circular chart depicting the collaborative landscape in AGA treatment research among the top 30 nations, derived from co‐authorship analysis. (D) A time overlay map showing the research output of the top 30 countries contributing to AGA treatment modalities, utilizing bibliographic coupling analysis.

According to our search, a total of 2203 studies on AGA treatment have been published over the past two decades. These publications demonstrate substantial research output on this topic. Between 2003 and 2019, the number of publications grew at a slow and relatively low rate (Figure 2A). However, from 2019 to 2021, the growth rate accelerated significantly, and although the publication growth rate slowed down from 2022 to 2023, it remained at a relatively high level (Figure 2A).

### 3.2 Countries

The analysis of 2203 publications reveals that the top 10 contributing countries are located in North America, Asia, and Europe. A visual analysis of these publishing countries highlights the most active nations in AGA research (Figure 2B,C). Over time, research hubs have gradually shifted from the USA to China (Figure 2D). Table 1 provides data on the number of publications, citations, and other relevant metrics for the top 10 countries in the field. The United States was the leading contributor, with 649 papers, accounting for 29.50% of the total (Table 1), underscoring the dominant role of the USA in advancing research in this area. China followed with 327 publications, representing 14.84%, highlighting its significant role in AGA research. Italy published 175 papers, South Korea 131, and Germany 123, further illustrating the active involvement of these countries in the field (Table 1). In terms of citations, the United States maintained a commanding lead with 21,203 citations, followed by Italy with 4689 and Canada with 4625. Although Canada ranked eighth in the number of publications, with only 96 articles, it had the highest average citation rate of 44.47%, indicating its considerable influence in the scientific community.

**TABLE 1:** Top 10 productive countries in the treatment of androgenetic alopecia.

Country	Publications	Citations	Average citation rate	% Of (Publications)
United States	649	21 203	32.67	29.50
China	327	4185	12.80	14.84
Italy	175	4689	26.79	7.94
South Korea	131	2747	20.97	5.95
Germany	123	4029	32.76	5.58
India	117	1528	13.06	5.31
United Kingdom	116	4590	39.57	5.27
Canada	104	4625	44.47	4.72
Spain	96	2761	28.76	4.36
Japan	89	1710	19.21	4.04

### 3.3 Institutions

**FIGURE 3:**
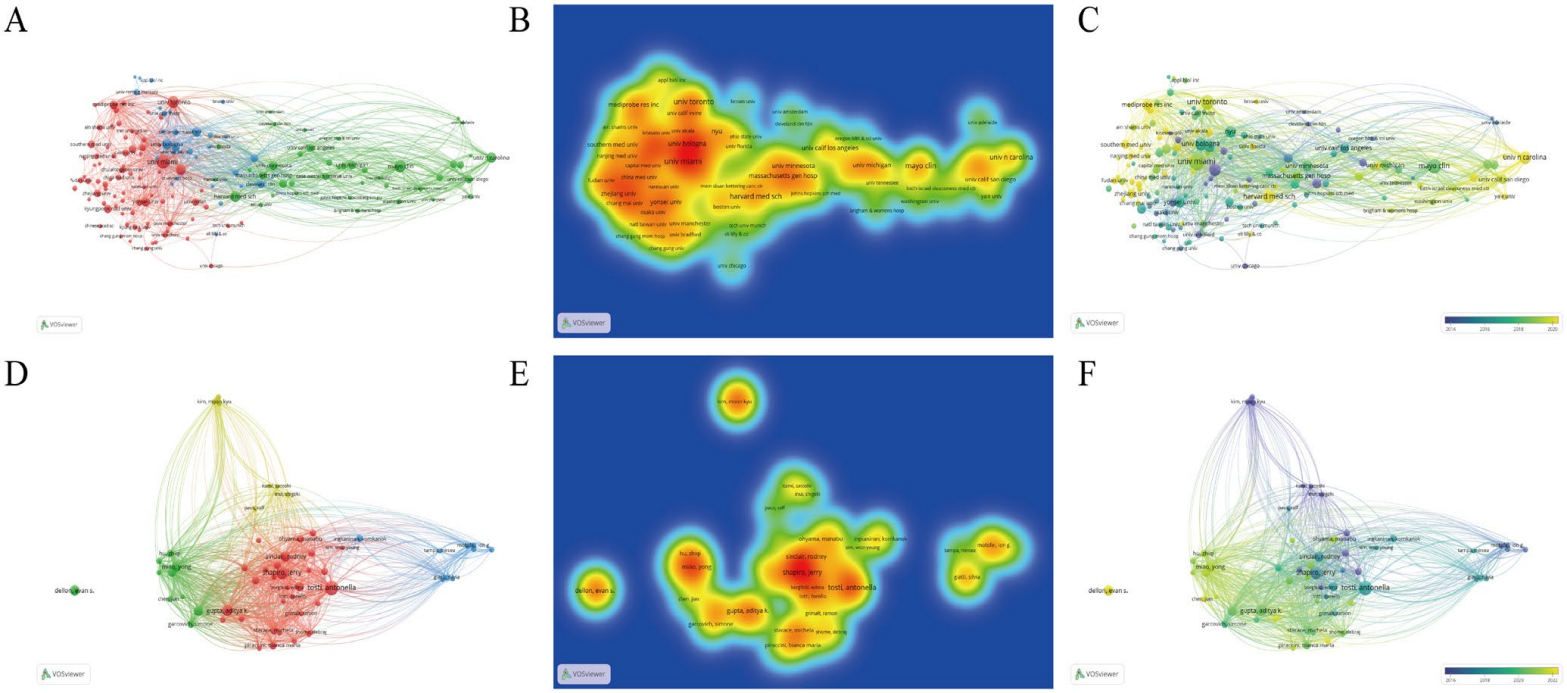
Enhanced global insights into the research landscape of AGA treatment. (A) An institutional network map illustrating the interconnectedness of studies on AGA treatment modalities, derived from co‐citation analysis. (B) A unit density map showcasing the geographic distribution of research institutions engaged in AGA treatment modalities, based on co‐citation analysis. (C) A time overlay of AGA treatment‐related research institutions. (D) A network diagram of authors actively contributing to the field of AGA treatment modalities, as revealed by co‐citation analysis. (E) An author unit density map highlighting the concentration of research efforts in various areas of AGA treatment, through co‐citation analysis. (F) A time overlay of authors in research areas related to AGA therapy.

Table 2 lists the top 10 institutions with the highest number of publications in AGA research. Half of these leading institutions are located in the Americas and North America (Table 2 and Figure 3A). At the top of the list is the University of Miami, with 49 published papers, followed by the University of Toronto with 39 papers, the Mayo Clinic with 39 papers, and Harvard Medical School with 33 papers. In terms of citations, the Mayo Clinic leads with 3009 citations, followed by the University of North Carolina with 1815 citations (Table 2 and Figure 3B). To gain deeper insights, we conducted further analysis. A year‐by‐year visual analysis was performed to examine the publication concentration of these institutions over time (Figure 3C). According to this analysis, Harvard Medical School and the University of North Carolina showed a higher concentration of publications in 2020.

**TABLE 2:** Top 10 institutions with the most publications in the treatment of androgenetic alopecia.

Institutions	Publications	Citations	Average citation rate
Univ Miami	49	1291	26.35
Univ Toronto	39	921	23.62
Mayo Clin	39	3009	77.15
Harvard Med Sch	33	737	22.33
Univ Bologna	32	1062	33.19
Univ N Carolina	32	1815	56.72
Univ British Columbia	31	1360	43.87
NYU	27	960	35.56
Univ Melbourne	27	514	19.04
Yonsei Univ	25	619	24.76

### 3.4 Authors

Analyzing the authors of academic literature is essential for identifying prominent scholars and core contributors in the field. Among the most prolific authors, Tosti Antonella stands out, having published 31 articles, which have been cited a total of 920 times (Figure 3D,E). Her publications are primarily concentrated around 2018 (Figure 3F). The next most prolific author is Shapiro Jerry, who has published 25 articles, accumulating 636 citations. Both authors have provided significant theoretical foundations for the advancement of AGA research.

### References

3.5

**FIGURE 4:**
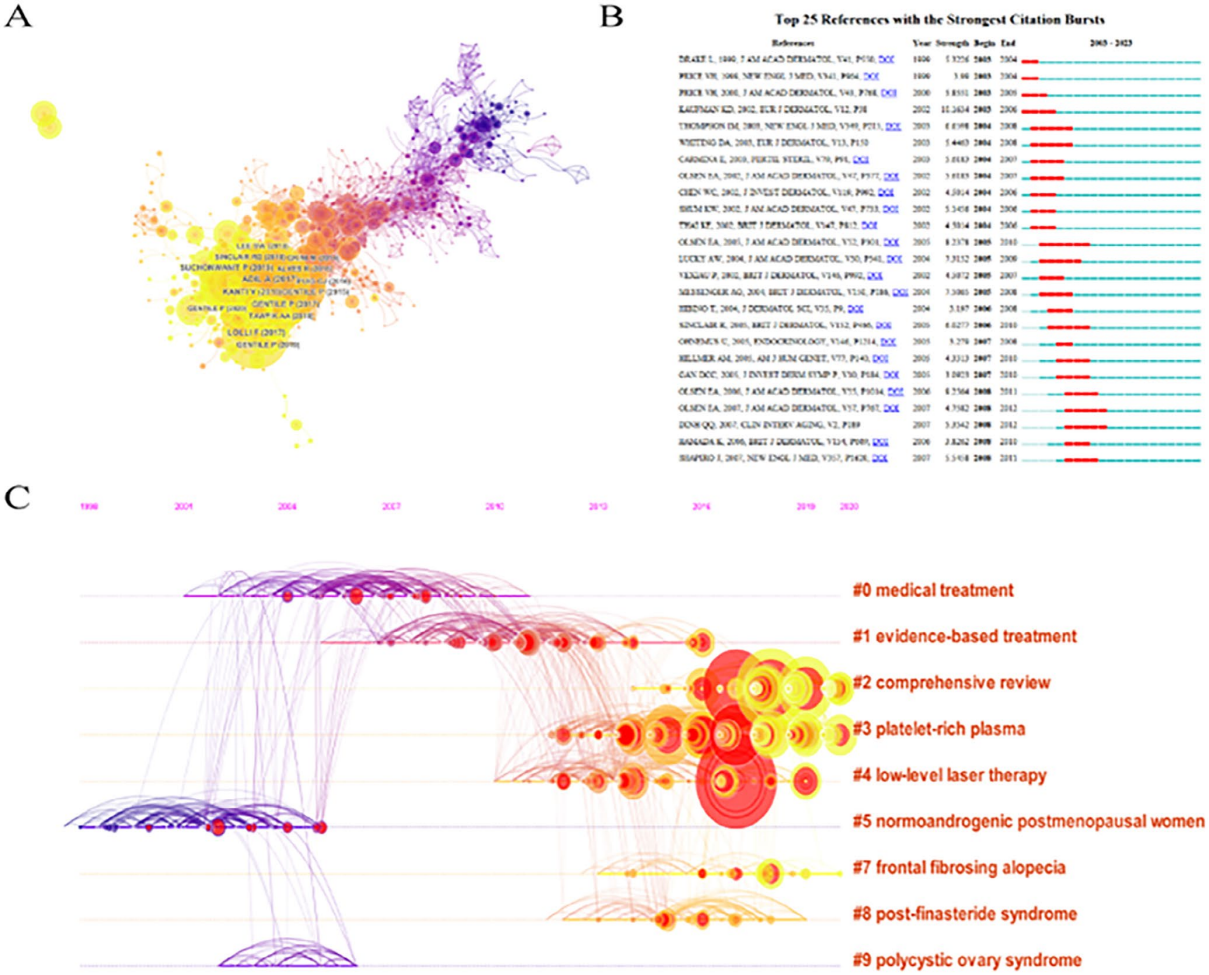
Global insights into the citation dynamics of AGA treatment research. (A) A co‐citation network map showcasing the interlinking of literature focused on AGA treatment modalities. (B) A timeline visualization that tracks the evolution of research clusters, as identified through the analysis of literature titles from 2003 to 2023. (C) A ranking of the top 25 most influential references, characterized by significant citation bursts within the field.

The top publications suggest that research in the field of AGA research has taken a new direction and remains a highly active topic. The publication “Androgen excess in women: experience with over 1000 consecutive patients” holds the highest number of citations (541). This study, published in the Journal of Clinical Endocrinology & Metabolism (impact factor = 5.80), underscores the effects of hyperandrogenism in women. A cluster analysis of literature titles from 2003 to 2023, visualized over time, provides a clearer understanding of hot research directions and the timing of research focus shifts (Figure 4B). The clustering timeline highlights the evolution of research focus, transitioning from early drug therapies to more recent treatments like anti‐androgen therapy, PRP therapy, and others (Figure 4B). Additionally, the top 25 most‐cited references offer valuable insights into emerging research trends and key topics (Figure 4C).

### 3.6 Keywords

**FIGURE 5:**
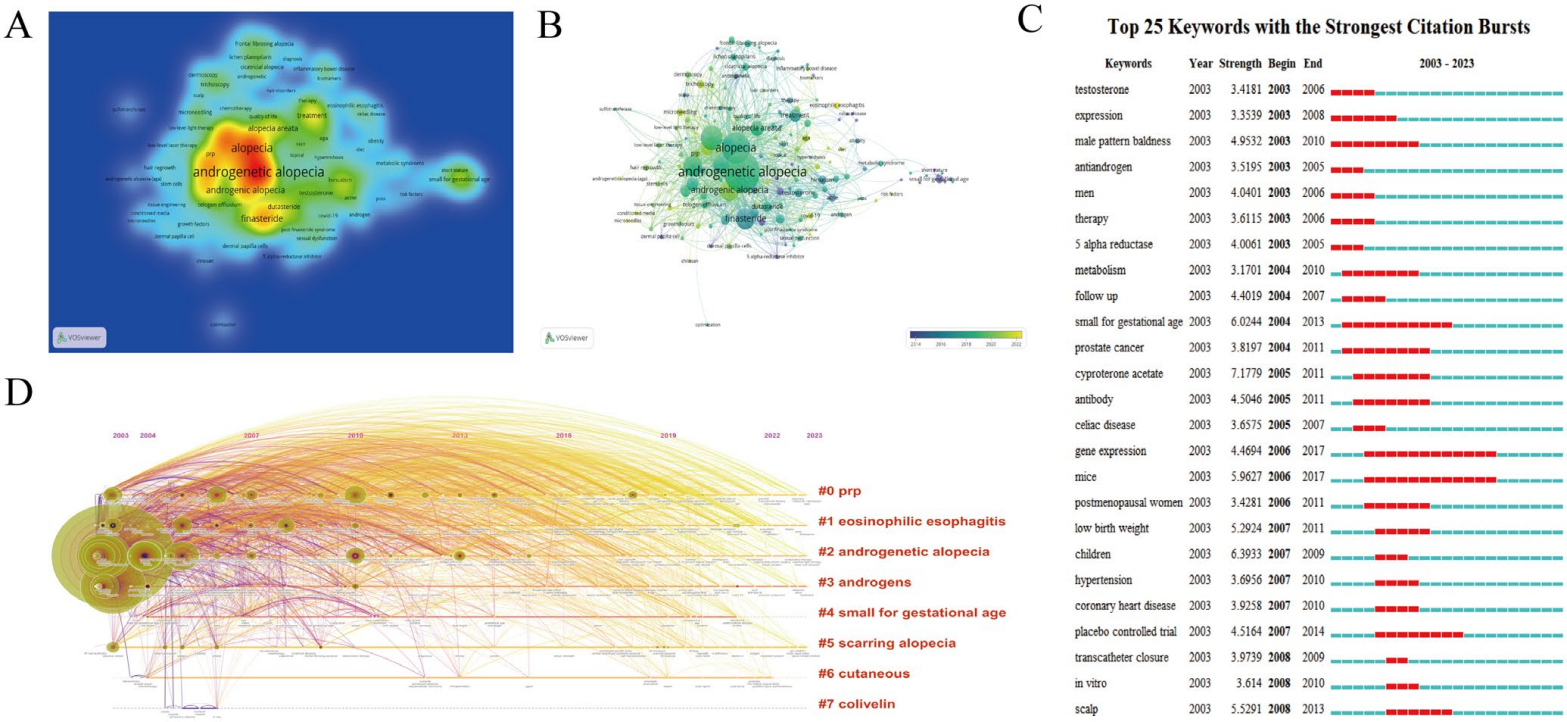
Global trends in keyword prominence within AGA research. (A) A unit density map highlighting the geographical distribution of keyword contributions to AGA research. (B) A time overlay chart illustrating the chronological emergence and prominence of AGA‐related research keywords. (C) A list of the top 25 keywords that have experienced the most significant surges in citations, indicating their impact on the field. (D) A timeline visualization depicting the results of cluster analysis on keywords, showcasing the thematic evolution of AGA research from 2003 to 2023.

Keywords play a crucial role in identifying the central theme of a publication. By analyzing keyword contributions, we can systematically understand the trending topics in AGA research, their internal relationships, and the burst concentration periods, as illustrated through color and size in Figure 5A,B. Among the keywords, “androgenetic alopecia (AGA)” stands out with the highest frequency and link strength. Additionally, the top 25 keywords with the strongest citation bursts are highlighted, reflecting various focus areas of development. Early keywords, such as PRP and androgens continued to garner significant attention (Figure 5C). Furthermore, the time overlay map reveals the evolving diversity within the AGA research field over time (Figure 5D).

## 4 Discussion

This study provides a comprehensive synthesis of the current research landscape and therapeutic advancements in AGA through bibliometric analysis. A total of 2203 studies were published between 2003 and 2023. As shown in Figure 5A, keywords, such as anti‐androgen, gene expression, and small for gestational age were prominent. From 2003 to 2008, the primary focus was on androgens, suggesting that understanding of AGA was still limited during this period. From 2008 to 2013, research expanded to include gene expression, cytokines, and animal model testing, indicating that the field had gained both depth and breadth (Figure 5D). From 2010 to 2020, the number of published papers increased significantly. During this decade, research directions focused on comprehensive reviews, PRP therapy, and laser therapy. Among these, PRP emerged as a novel method for treating AGA, providing a theoretical foundation and demonstrating promising clinical results. Both China and the USA have made substantial contributions to AGA therapy research. The USA, in particular, has been a leading contributor, with 649 articles published and 21 203 citations (Table 1). More than half of the top 10 institutions with the most publications are located in the USA, with the Mayo Clinic having an average citation rate of 77.15. These high‐quality journals and institutions play a crucial role in supporting advancements in AGA treatment. Regarding authorship, Tosti Antonella stands out as the most prolific author, having published 31 papers with a total of 920 citations (Figure 3D,E). Antonella's extensive research on hair loss treatments has made significant contributions to the field. To gain insights into the latest trends in AGA treatment, we conducted a literature title cluster analysis and a keyword co‐occurrence cluster analysis (Figures 4B and 5C). These analyses provide a clearer picture of the current hot topics and main research directions, enhancing our understanding of the field. Based on these findings, we can identify the following hot areas of research.

### 4.1 Anti‐Androgen Therapy

Androgens play a crucial role in the development of AGA, with elevated levels leading to impaired microcirculation in the scalp [8]. Oral finasteride is a well‐established treatment for male pattern baldness, working by inhibiting the type 2 5‐alpha reductase enzyme, thereby preventing the conversion of testosterone to dihydrotestosterone (DHT) and reducing androgen‐mediated follicular miniaturization [9]. Although finasteride's effectiveness is well‐documented, it is important to recognize potential side effects, which may include altered sexual desire, erectile dysfunction, ejaculatory disorders, and in some cases, depression and suicidal ideation [10].

Recent studies have introduced novel topical treatments for AGA, including the androgen receptor inhibitors Clascoterone and Pyrilutamide. Clascoterone, a pioneering topical androgen receptor inhibitor, was approved in 2020 for the treatment of acne in patients aged 12 and older [11]. Due to its anti‐androgen properties, Clascoterone is also believed to offer potential benefits for the treatment of AGA. Research has shown that Clascoterone can effectively increase hair diameter and follicular density [12]. Additionally, it is generally well‐tolerated, with the main adverse effects being mild dryness, erythema, and increased hair growth at the application site [11]. Pyrilutamide, another innovative topical androgen receptor inhibitor, has also shown promise. In a Phase II clinical trial, Pyrilutamide demonstrated efficacy in treating male pattern hair loss and exhibited a favorable safety profile [13].

For the oral treatment of AGA, current medications primarily include finasteride for men and spironolactone for women. Low‐dose oral minoxidil (≤ 5 mg/day) is gaining increasing attention for its efficacy in treating hair loss. Studies have demonstrated that this low dose of oral minoxidil can improve both male and female pattern hair loss [14, 15]. Minoxidil enhances the expression of Vascular Endothelial Growth Factor (VEGF) [16], stimulates hair growth by activating prostaglandin oxidizing cyclase, and extends the anagen phase by activating the beta‐catenin pathway. Common concentrations for topical minoxidil are 2% and 5%, with the consensus that a 5% topical application tends to produce superior results [17, 18, 19, 20]. Another promising treatment is bicalutamide, a novel oral non‐steroidal androgen receptor inhibitor. In a retrospective study of 17 female AGA patients treated with 50 mg of bicalutamide daily or every other day for 24 weeks, 57% of patients showed significant hair regrowth [21]. In terms of side effects, a study involving 316 participants reported that 9 individuals experienced a mild increase in liver enzyme levels, though 4 of these cases returned to normal without any dosage adjustments [22].

### 4.2 Platelet‐Rich Plasma

Among the emerging treatments for AGA, PRP therapy has gained popularity due to its high efficacy, minimal side effects, and low recurrence rates. Numerous studies involving AGA patients have demonstrated that PRP therapy can significantly increase hair density and diameter [23, 24]. A recent study also investigated the effects of PRP therapy on the scalp microbiota, revealing significant improvements and positive outcomes after treatment and analysis of scalp samples [4]. However, the effectiveness of PRP is dependent on the integrity of the hair follicles, making it more effective for early‐stage AGA patients whose follicles have not yet been fully damaged.

Currently, there is no standardized protocol for the preparation of PRP, although the general process involves centrifugation of the patient's own blood to obtain the concentrated platelet‐rich plasma. Potential side effects include scalp pain, pinpoint bleeding, headache, and a burning sensation, which typically subside within 10–15 min post‐injection. Most patients do not require local or oral analgesics for these minor effects and can resume normal activities, although it is recommended to avoid strenuous physical activities for 24 h after treatment. Patients with a history of bleeding disorders, active infections, or autoimmune diseases should avoid PRP therapy due to increased risks [25].

### 4.3 Botulinum Toxin A (BTA)

In recent years, local injections of BTA have gained popularity as a treatment for AGA due to their ability to counteract the inhibitory effects of DHT on hair follicles. BTA also relaxes local muscles and improves scalp blood circulation, enhancing the supply of oxygen and nutrients to the scalp, thereby promoting hair growth [26]. In another randomized controlled trial involving 37 patients with androgenetic alopecia, it was found that the combination therapy of BTA injections with oral finasteride and minoxidil was more effective than oral finasteride and minoxidil alone, with no significant side effects observed [27].

It is noteworthy that local BTA injections are suitable for both male and female patients, providing a new perspective on AGA treatment. However, the precise mechanism of action is not yet fully understood and requires further investigation. Additionally, the long‐term therapeutic effects of BTA in patients need to be monitored for a more comprehensive evaluation.

### 4.4 Microneedle Therapy

Microneedle therapy is a minimally invasive dermatological procedure that has been used to treat a variety of skin conditions, including AGA. The technique involves rolling fine needles over the skin to puncture the stratum corneum, creating micro‐perforations that stimulate the release of platelet‐derived growth factors and VEGF. This process promotes angiogenesis, wound healing, and the reversal of fibrosis at the treatment site. Another potential mechanism is the creation of channels through these micro‐wounds, which may enhance the absorption of topical treatments such as minoxidil or finasteride at concentrations of 2%–5% [28].

There are reports suggesting that microneedling shows promise in improving hair growth, especially when combined with existing therapies. In a review of 17 studies involving a total of 911 AGA patients, significant improvements in hair parameters were observed when microneedling was combined with 5% minoxidil, growth factor solutions, and/or PRP [29].

### 4.5 Stem Cell Therapy

Research has shown that stem cells can promote the regeneration of hair follicles in the skin [16]. Regenerative pluripotent stem cells are primarily derived from adipose tissue, bone marrow, hair follicles, and umbilical cord tissue [19]. Adipose‐derived stem cells (ADSCs), which are easily obtained from subcutaneous and other fat tissues, are frequently used for experimental analysis [20]. ADSCs secrete a variety of bioactive molecules, including growth factors that influence surrounding cells and regulate the microenvironment necessary for hair follicle growth, playing a crucial role in the formation of new blood vessels. Early injection of autologous ADSCs into the scalp during AGA treatment can enhance the vascular distribution in the scalp and improve the survival rate of transplanted hair [17]. Due to ongoing efforts by researchers, stem cell therapy has gradually become a hot topic in AGA treatment and is expected to be a key focus for optimizing future treatment methods [18].

### 4.6 Genetic and Molecular Mechanism Studies

Numerous studies worldwide have shown that AGA has a genetic predisposition involving multiple genes and factors. Experts have identified over 250 independent genetic loci potentially related to the severity of hair loss, developed a prediction algorithm based entirely on common genetic variants, and even estimated the potential costs associated with AGA [30]. Numerous studies have demonstrated that genetic predisposition plays a significant role in the etiology of androgenetic alopecia. Recent genome‐wide association studies on AGA have revealed significant association signals within the X chromosome. Notably, both the androgen receptor (AR) gene and the ectodysplasin A2 receptor (EDA2R) gene, located at the AR/EDA2R locus in the Xq11‐q12 region, exhibited strong genetic associations with AGA [31]. In fact, genetic studies of AGA primarily focus on androgen and its receptor. The AR gene, located on Xq11.2‐q12 and approximately 90 kb in length, was the first gene associated with AGA [32]. Since AGA predominantly affects men, the Y chromosome, which determines sex and regulates sex hormone distribution, plays a key role. However, the pseudoautosomal region at the end of the Y chromosome, which can recombine with the X chromosome, has been studied less extensively.

## 5 Conclusion

This bibliometric study provides a comprehensive overview of the AGA treatment landscape, highlighting significant advancements and identifying key areas for future exploration. By meticulously analyzing two decades of scholarly work, we have traced the evolution of AGA treatments from traditional approaches to innovative therapies. Our findings emphasize the need for continued research, particularly in the burgeoning fields of stem cell and gene therapies, which hold the potential to revolutionize AGA management. It should be noted that the use of generic abbreviations in the search strategy may have led to a small number of cross‐disciplinary records being included, which could marginally affect the lists of highly cited publications and keyword clusters; however, these factors do not alter the core conclusions regarding the overall research landscape and its evolutionary trajectory.

In conclusion, the dynamic field of AGA research is poised for further investigation, with the potential to reveal more effective, safer, and longer‐lasting solutions. This study serves as a guiding resource for researchers, clinicians, and stakeholders, illuminating the trajectory of AGA treatment and reinforcing the collective effort to improve patient care and outcomes.

## Author Contributions

Y.S. and X.C. had full access to all the data in the study and take responsibility for the integrity of the data and the accuracy of the data analysis. Data acquisition, processing, and analysis were conducted by Y.S., M.L., X.G., and Y.L., with J.R. and Y.W. leading result interpretation. Y.S. and M.L. drafted the manuscript, while X.C., Y.L., and J.R. revised the text for clarity and scientific rigor. All authors reviewed and approved the final version.

## Ethics Statement

The authors have nothing to report.

## Conflicts of Interest

All authors states that the research was conducted in the absence of any commercial or financial relationships that could be construed as a potential conflicts of interest.

## Data Availability

Data will be made available on request.
